# Pleural Effusion in a Patient With Chronic Myelomonocytic Leukemia Treated With Azacitidine

**DOI:** 10.7759/cureus.78004

**Published:** 2025-01-26

**Authors:** Marwa Mir, Bella Gnakou, Akila Gill, Jia Yi Tan, Wajeeha Aiman, Hamid S Shaaban, Gunwant Guron

**Affiliations:** 1 Internal Medicine, Saint Michael's Medical Center, Newark, USA; 2 Internal Medicine, Morristown Medical Center, Morristown, USA; 3 Hematology and Oncology, Saint Michael's Medical Center, Newark, USA

**Keywords:** chronic myelomonocytic leukemia (cmml), extramedullary hematopoiesis, myelodysplastic neoplasm, pleural effusion treated with azacitidine, tet2

## Abstract

Pleural effusion is an uncommon occurrence in chronic myelomonocytic leukemia (CMML) patients, and its mechanisms remain poorly understood. We report the case of a 66-year-old male with a known medical history of CMML, referred from the oncology clinic due to shortness of breath attributed to a left pleural effusion, evident on a chest X-ray following a chemotherapy session. A diagnostic thoracentesis and cytology were conducted, which yielded exudative fluid negative for malignant cells and found reactive mesothelial cells and macrophages in a background of numerous chronic inflammatory cells and acellular proteinaceous material. However, due to the limited effectiveness of cytological examination in identifying malignant pleural effusions, the occurrence of leukemic effusions in CMML patients may be underestimated. This case underscores the importance of prompt recognition and management of pleural effusion in patients with underlying hematologic conditions like CMML.

## Introduction

Pleural effusion can manifest among hematological malignancies due to various disease-related factors or treatment-related complications. While relatively uncommon in chronic myelomonocytic leukemia (CMML), pleural effusion can occur through mechanisms such as leukemic infiltration into the pleura or extramedullary hematopoiesis (EMH), where abnormal blood cell production occurs outside the bone marrow [1]. Additionally, certain chemotherapy agents used in CMML treatment, like tyrosine kinase inhibitors (TKIs), may contribute to pleural effusion formation as a side effect, underscoring the multifaceted nature of this condition in leukemia patients [2].

## Case presentation

**Figure 1:**
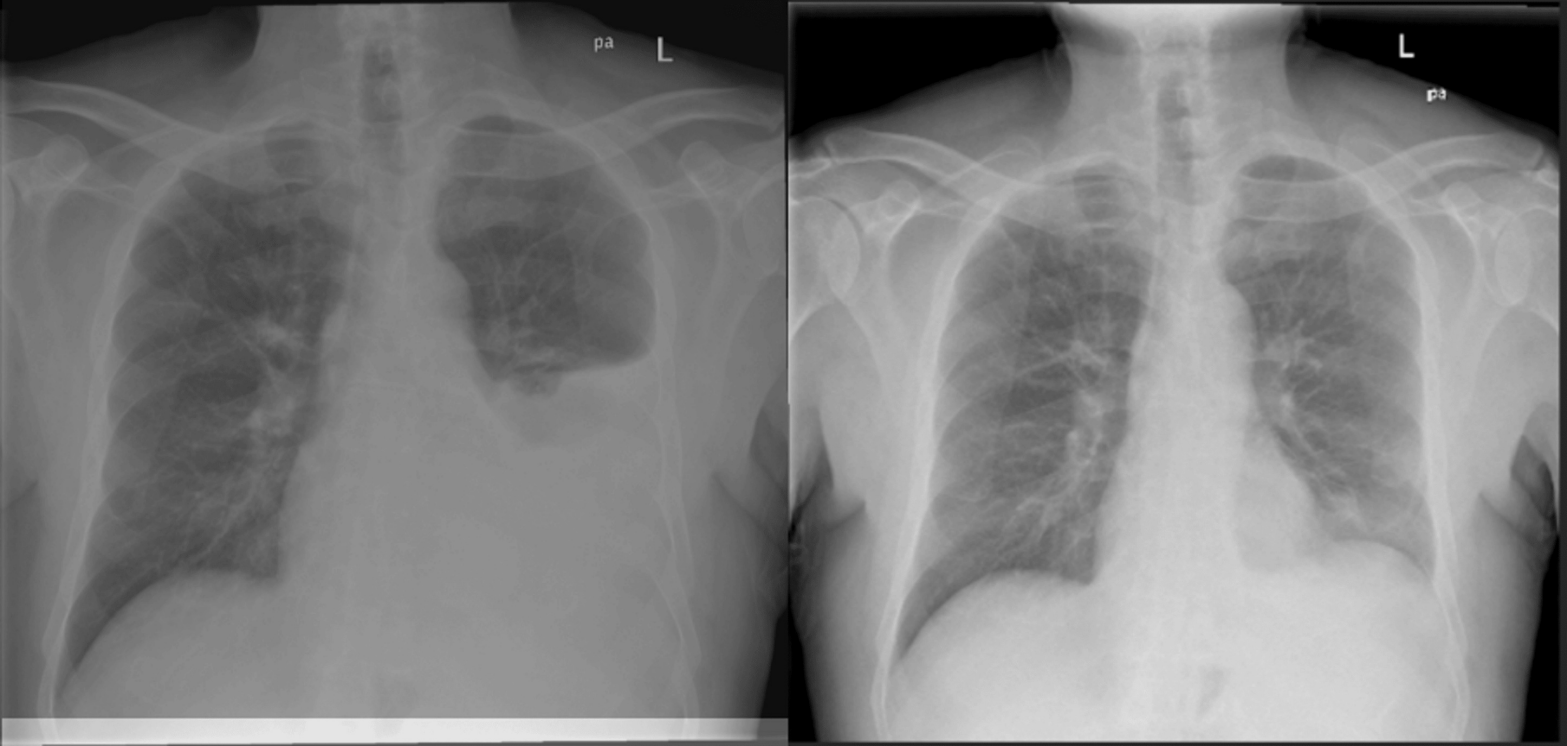
Two-view examination of the thorax on the left showing a large left pleural effusion reaching the superior aspect of the left hilum. Complete silhouetting of the left heart border and left hemidiaphragm is consistent with consolidation and atelectatic change within the lingula and left lower lobe. There is some compressive atelectasis in the left upper lobe. There is no pneumothorax. The image on the right depicts the post-thoracentesis result.

The patient is a 66-year-old male with a medical history notable for recently diagnosed CMML, recurrent splenic infarction, and hypothyroidism. Presented with complaints of shortness of breath attributed to left-sided pleural effusion. This was evident on a chest X-ray performed following a chemotherapy session (Figure 1). He denied experiencing abdominal pain, fever, or nausea. Bone marrow aspiration was conducted after the second visit to the hospital with abdominal pain and an interval increase in spleen size at that admission. A blood smear revealed normocytic normochromic anemia, thrombocytopenia (76), and absolute monocytosis was noted. The bone marrow was found to be hypercellular with marked megakaryocyte hyperplasia with dysmegakaryopoiesis with loose cluster formation. CD34+ blasts are not increased. Previous testing showed trisomy 8 by karyotype and fluorescence in situ hybridization (FISH). No JAK2, CALR, or MPL mutations were detected. The findings are consistent with a myelodysplastic/myeloproliferative neoplasm, such as CMML. Next-generation sequencing revealed a 10-11 translocation oncogene family member 2 (TET2) mutation.

Physical examination revealed stable vital signs and an afebrile state. Pulmonary effort was observed to be expected, although tachypnea was present. Auscultation of the left-middle and left-lower lung fields revealed decreased breath sounds. Cardiovascular and central nervous system examinations were unremarkable. The clinical impression pointed toward a substantial left-sided pleural effusion.

The patient underwent diagnostic and therapeutic thoracentesis, draining 1500 cc of serosanguinous fluid. Pleural fluid analysis showed protein 4.5, glucose 129, and lactate dehydrogenase (LDH) 427, confirming an exudative effusion. Subsequent repeat X-rays demonstrated resolution of the pleural effusion, with no evidence of pneumothorax or consolidation. A repeat X-ray demonstrated the resolution of the pleural effusion without evidence of pneumothorax or consolidation. Following thoracentesis, the patient reported relief from shortness of breath and improved clinical status. A plan was devised for pleural biopsy to explore potential pleural involvement further. The patient was initially treated with hydroxyurea but developed severe cytopenia and gastrointestinal adverse effects. Additionally, the patient was cleared to continue vidaza injections as part of his ongoing chemotherapy treatment protocol. He tolerated the first cycle well with lactate dehydrogenase, and his complete blood count is improving.

## Discussion

CMML is a myelodysplastic neoplasm characterized by the proliferation of abnormal myeloid and monocytic cells in the bone marrow and peripheral blood. Diagnostic criteria are monocyte (≥1x10^9^/L), with monocytes comprising ≥10% of the total white blood cell count, leading to various systemic manifestations [3]. While less commonly associated with pleural effusion compared to other hematological malignancies, such as chronic myeloid leukemia (CML), CMML can still give rise to this complication through similar underlying mechanisms [4].

Similar to CML, CMML can lead to the infiltration of leukemic cells into extramedullary tissues, including the pleura. The infiltration of abnormal myeloid and monocytic cells into the pleural space can disrupt normal pleural fluid dynamics, resulting in fluid accumulation and pleural effusion formation [4,5]. In some cases of CMML, EMH may occur as a compensatory response to bone marrow dysfunction. Hematopoietic cells, including abnormal myeloid and monocytic precursors, may proliferate outside the bone marrow, potentially involving the pleura [6]. This aberrant hematopoiesis can contribute to the development of pleural effusion in CMML patients. Monocytes, a key component of CMML, are known to play a role in mediating inflammatory responses. Dysregulated activation of monocytes and their secretion of pro-inflammatory cytokines, such as tumor necrosis factor-alpha (TNF-alpha) and interleukin-6 (IL-6), may lead to increased vascular permeability and inflammation within the pleural space [7]. This inflammatory response can result in fluid leakage into the pleural cavity and form pleural effusion.

In advanced stages of CMML, myelofibrosis may develop, leading to the disruption of normal hematopoiesis. As bone marrow function deteriorates, extramedullary sites, including the pleura, may become increasingly involved in hematopoiesis [8]. Expressing hematopoietic tissue in the pleura can contribute to pleural effusion formation. Myelofibrosis may also lead to splenomegaly and portal hypertension, increasing the risk of ascites and pleural effusion due to elevated systemic venous pressure and impaired lymphatic drainage [7,8].

CMML patients commonly have TET2 mutations. The majority of the TET2 mutations in CMML are frameshift and nonsense mutations [9]. Previous studies in 2009 have shown TET2 mutation to be a favorable independent prognostic factor in myelodysplastic syndrome (MDS), and TET2 mutations were associated with poor outcomes in CMML patients [10]. Serositis or pleural effusion can result from inflammation present in the cells. Particularly in CMML, TET2 mutations lead to serositis through autoimmune manifestations and due to upregulated cytokine production. Some treatment modalities used in CMML management, such as chemotherapy or hypomethylating agents, may predispose patients to the development of pleural effusion as a side effect. Dasatinib, a second-generation TKI approved for treating CML, has been associated with a 5% to 15% risk for pleural effusion per year [11]. Additionally, blood transfusions, which are commonly administered to CMML patients, can lead to volume overload and exacerbate fluid accumulation in the pleural cavity. In such cases, demethylating agents may be the preferred treatment for serositis [12].

While the cytologic examination in this case pointed to an exudative fluid, cytopathology often has limited diagnostic value in hematologic malignancies due to its low potential for accurate diagnosis, with reported sensitivity between 40% and 90% [10]. In such cases, pleural biopsy is the gold standard in diagnosing a malignant pleural effusion. Clinicians must recognize this uncommon progression of CMML to prevent misdiagnosis.

## Conclusions

Despite the rarity of pleural effusion in CMML, clinicians should maintain a high index of suspicion for such complications, especially in patients with a known history of CMML. Pleural effusion in CMML, although rare, can be critical in diagnosis and treatment. Diagnostic challenges, including the limited utility of cytology, underscore the need for comprehensive diagnostic evaluations, such as pleural biopsies, to ensure accurate diagnosis and appropriate management. For cases with pleural effusion, CMML should be at least in the differential diagnosis. TET2 mutations have significance in MDS and CMML clinical pathogenesis. Although the prognosis correlation is not well established. The choice of treatment for CMML patients with pleural effusion depends on the risk stratification that includes age, comorbidities, and side effect profiles. Demyelinating agents are preferred agents over intensive chemotherapy. Steroids are also employed as a treatment for pleural effusion. In patients with lower-risk CMML, allogeneic hematopoietic cell transplantation can cure CMML but has substantial toxicity. Hydroxyurea can also be used to provide symptom relief. In this case, since the patient developed severe cytopenia and GI side effects on hydroxyurea, the patient was started on vidaza, which showed improvement in LDH and CBC. The treatment successfully prevented the re-accumulation of pleural effusion in the patient.
